# Supplementary material for: Domino-like effect of C112R mutation on ApoE4 aggregation and its reduction by Alzheimer’s Disease drug candidate
Source: Mol Neurodegener. 2023 Jun 6;18:38. doi: 10.1186/s13024-023-00620-9 (PMC10245438; doi:10.1186/s13024-023-00620-9)
Supplement: Supplementary file 3 — Supplementary Material 3 [file 13024_2023_620_MOESM3_ESM.pdf]

Drs. Martin Marek, David Bednar, Jiri Damborsky
Loschmidt Laboratories, RECETOX Institute
Faculty of Science, Masaryk University
Kamenice 753/5 Bld. C13, 625 00 Brno, Czech Republic
Phone: 420-5-49498763, Fax: 420-5-49496302
Email: martin.marek@recetox.muni.cz

---

April 12, 2023

**Dear Editor Prof. Dr. David Holtzman,**

Please find enclosed a revised version of the research paper entitled: "**Domino-like Effect of C112R Mutation on ApoE4 Aggregation and Its Reduction by Alzheimer's Disease Drug Candidate**", which we are submitting for re-consideration by the *Molecular Neurodegeneration.*

We want to express our thanks to you and the referee for constructive and highly relevant comments. We did our best to incorporate his/her suggestions in our revised manuscript. We have also conducted a point-by-point response to all comments/concerns raised by the referee and revised all the manuscript parts, which were not sufficiently clear in the previous version. We hope that these changes will make our manuscript acceptable for publication in *Molecular Neurodegeneration.*

Sincerely yours,

Martin Marek, David Bednar and Jiri Damborsky
on behalf of the co-authors
